# Heterologous Expression of Sunflower *HaHPT* and *HaTMT* Genes Enhances Rice-Grain Vitamin E Content

**DOI:** 10.3390/plants13172392

**Published:** 2024-08-27

**Authors:** Shuang Song, Hang Li, Shaoyan Lin, Xiaoou Dong, Ruiping Tian, Zewan Wu, Qing Li, Mingyi Li, Keying Zhang, Xi Liu, Jianmin Wan, Linglong Liu

**Affiliations:** 1 State Key Laboratory for Crop Genetics & Germplasm Enhancement and Utilization, Jiangsu Nanjing National Field Scientific Observation and Research Station for Rice Germplasm, Key Laboratory of Biology, Genetics and Breeding of Japonica Rice in Mid-Lower Yangtze River, Ministry of Agriculture and Rural Affairs, Sanya Research Institute, Nanjing Agricultural University, Nanjing 210095, China; 2 Zhongshan Biological Breeding Laboratory, Nanjing 210095, China; 3 State Key Laboratory of Crop Gene Resources and Breeding, Institute of Crop Sciences, Chinese Academy of Agricultural Sciences, Beijing 100081, China

**Keywords:** rice (*Oryza sativa* L.), vitamin E, sunflower (*Helianthus annuus* L.), homogentisate phytyltransferase, γ-tocopherol methyltransferase

## Abstract

Insufficient dietary vitamin intake can lead to severe health conditions in humans. Improving the vitamin E (VE) content of food crops such as rice through breeding is an economical and effective means to alleviate this problem. In this study, *Homogentisate phytyltransferase* (*HPT*) and *γ-tocopherol methyltransferase* (*γ-TMT*), two genes derived from sunflower (*Helianthus annuus* L., a high VE species), were introduced into an elite rice (*Oryza sativa* L.) cultivar “Ningjing 7” for biofortification. We verified the successful expression of the two genes in multiple transformation events. High-performance liquid chromatography revealed that transgenic plants expressing either *HaHPT* alone or *HaHPT* and *HaTMT* accumulate more VE compared with the wild type. We also revealed that the level of α-tocopherol, the form of VE with the highest biological activity, had increased to 2.33 times in transgenic *HaTMT* plants compared with the wild type. Transcriptome analysis revealed that the expression levels of some chlorophyll synthesis pathway genes related to VE precursor synthesis significantly increased during grain filling in transgenic rice grains. No difference in agronomic traits was observed between the transgenic plants and their wild type except for a slightly reduced plant height associated with the transgenic plants. These data demonstrate that the heterologous expression of *HaHPT* gene is effective in increasing the total VE content, while *HaTMT* plays an important role in the relative abundance of α-tocopherol in rice grains. This study demonstrates a promising strategy for breeding rice with elevated VE content via metabolic engineering.

## 1. Introduction

Rice (*Oryza sativa* L.) a major staple food crop, fulfilling the calorie needs of more than half of the world population. With the improvement of people’s living standards, the public’s demand for the quality of rice is increasing, especially the nutritional quality of rice [1]. Hidden hunger is a common problem in developing countries and underdeveloped areas, caused in part by the insufficient uptake of trace nutritional elements such as vitamins. Although the problem of ‘hidden hunger’ can be alleviated by diversified diets, the cost is high and it is difficult to implement in large areas in developing countries or underdeveloped areas [2,3]. As an essential fat-soluble vitamin, vitamin E (VE) is essential for human health and cannot be synthesized in the human body; thus, it can only be acquired through dietary intake [4,5]. However, the content of VE in rice grains is low, which makes it difficult to meet the daily needs of the human body. Therefore, the biofortification of VE in rice is of great significance for improving the nutritional quality of rice [6,7].

According to the side chain structure, VE can be divided into two groups. The side chain group that is saturated is called tocopherol, and the unsaturated group is called tocotrienol. According to the difference in the number and position of methyl on the aromatic ring, VE can be divided into four types: α, β, γ, and δ. Among them, α-tocopherol has the highest biological activity and is easily absorbed by humans and animals [8]. The content of VE in different plants or different tissues of the same plant varies greatly. α-tocopherol is the major isomer in sunflower (*Helianthus annuus* L.) seeds. Wheat (*Triticum aestivum*) grains contain mainly α- and β-type isomers, with β-tocotrienol being the major isomer. Barley (*Hordeum vulgare*) seeds contain all isomers, and the major isomer is α-tocotrienol [9]. Rice seeds contain mainly α- and γ-type isomers, and γ-tocotrienol is the major isomer [10]. In fact, studies have shown that sunflower has the highest α-tocopherol content among common plant oilseed species [11].

Homogentisate phytyltransferase (HPT) is a key rate-limiting enzyme for tocopherol synthesis. It catalyzes the condensation of homogentisate (HGA) and phytylpyrophosphate (PPP) to form 2-methyl-6-phytylbenzoquinol (MPBQ), thereby affecting downstream tocopherol synthesis. In *Arabidopsis* (*Arabidopsis thaliana*), *VITAMIN E 2* (*VTE2*) was identified to encode an HPT (At2g18950). Seed development in the *Arabidopsis vte2* mutant is poor, and the tocopherol component drops below detectable levels [12]. Overexpression of the *AtHPT* gene in *Arabidopsis* resulted in a two-fold increase in seed tocopherol content, while the content of α-tocopherol and γ-tocopherol in the leaves of *HPT* transgenic lettuce (*Lactuca sativa*) increased by 4 times and 2.6 times, respectively [13]. Under the action of γ-tocopherol methyltransferase (γ-TMT), δ-tocopherol and γ-tocopherol are converted to β-tocopherol and α-tocopherol, respectively. As the last key rate-limiting enzyme in the VE synthesis pathway, *γ-TMT* was first discovered in pepper (*Capsicum annuum*) [14]. After the transformation of the *γ-TMT* gene in alfalfa (*Medicago sativa*), the content of α-tocopherol increased, and the senescence of leaves was delayed [15]. The overexpression of *γ-TMT* in *Arabidopsis* and rapeseed (*Brassica napus*) also promoted the accumulation of α-tocopherol and methyl PC-8 in the seeds [16]. Likewise, the overexpression of *γ-TMT* in soybean (*Glycine max*) increased α-tocopherol content [17]. Although the overexpression of *HPT* and *γ-TMT* has been reported in *Arabidopsis* and other plants to result in increased VE content [18,19], their heterologous expression in rice has not been attempted.

In this study, *HaHPT* and *HaTMT* from sunflower were overexpressed in the elite *japonica* rice variety Ningjing 7. The relative abundance of total VE content in the dehusked rice grain (brown rice) from *HaTMT* transgenic plants had increased by 18.23%, while that of *HaHPT* transgenic plants had increased by 33.26% compared to the control samples. At the same time, the transgenic plants expressing both *HaTMT* and *HaHPT* showed the most evident increase, which had 35.98% higher VE content than the wild type. The VE-enriched rice germplasms developed in this study provide the basis for improving the nutritional quality of rice. 

## 2. Results

### 2.1. HPLC Analysis of Vitamin E Isomers 

**Figure 1:**
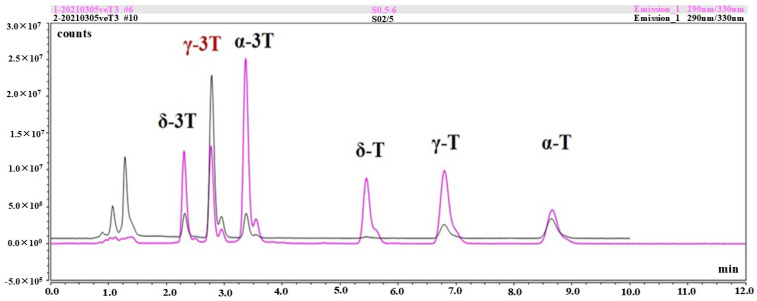
The order of peaks in the HPLC assay corresponding to various vitamin E (VE) components. The peak from left to right: δ-3T, δ-tocotrienol; γ-3T (in brown), γ-tocotrienol; α-3T, α-tocotrienol; δ-T, δ-tocopherol; γ-T, γ-tocopherol; α-T, α-tocopherol. The purple line represents the standards of various VE components, while the black line indicates the chromatography curve of the measured sample isolated from dehusked rice grains.

In the determination of VE in rice grains by ultra-high-performance liquid chromatography (HPLC), the total peak time of all isomers in each sample was about 12 min. The order of peaks of VE isomers were δ-tocotrienol, γ-tocotrienol, α-tocotrienol, δ-tocopherol, γ-tocopherol, and α-tocopherol, and the proportions of each isomer were 7.77%, 56.40%, 7.59%, 1.00%, 11.67%, and 15.57%, respectively (Figure 1). Due to the low percentages for the δ-tocopherol and δ-tocotrienol, the contents of β-tocopherol and β-tocotrienol (the catalyzed products from δ-tocopherol and δ-tocotrienol) were below detectable levels in rice seeds. We also found that the peak time of each isomer in different sample batches occasionally varied due to changes in instruments and environment (such as buffer, temperature, voltage, etc.), but on the whole, the peak time of each isomer did not differ much from the VE standards, so our protocol could accurately identify different VE isomers in rice grains.

### 2.2. Acquisition and Identification of Transgenic Plants

**Figure 2:**
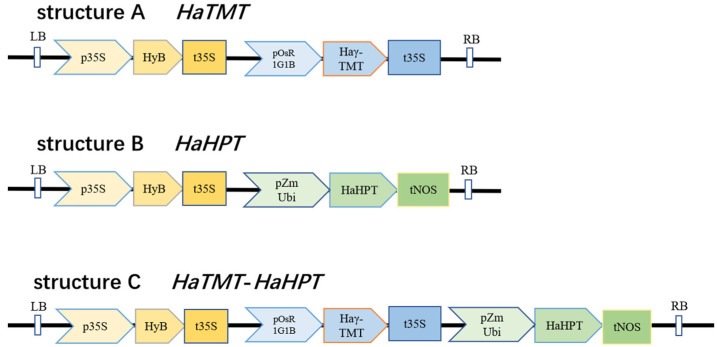
Schematic diagram of single-gene and double-gene co-transformation vectors. Structure A is the schematic diagram of *γ-tocopherol methyltransferase* (*γ-TMT*) transgenic construct. Structure B is the schematic diagram of *Homogentisate phytyltransferase* (*HPT*) transgenic construct. Structure C is the schematic diagram of *HaTMT* and *HaHPT* double-gene construct. LB: Left border. RB: Right border. *p35S*: *CaMV35S* promoter. *HyB*: The gene encodes hygromycin B phosphotransferase. *pOsR1G1B*: Constitutive strong promoter encodes early drought-inducible protein. *pZmUbi*: Constitutive strong promoter Ubiquitin. *HaHPT*: Sunflower *HPT* gene. *HaTMT*: Sunflower *γ-TMT* gene. *t35S*: 35S terminator. *tNOS*: *NOS* terminator.

**Figure 3:**
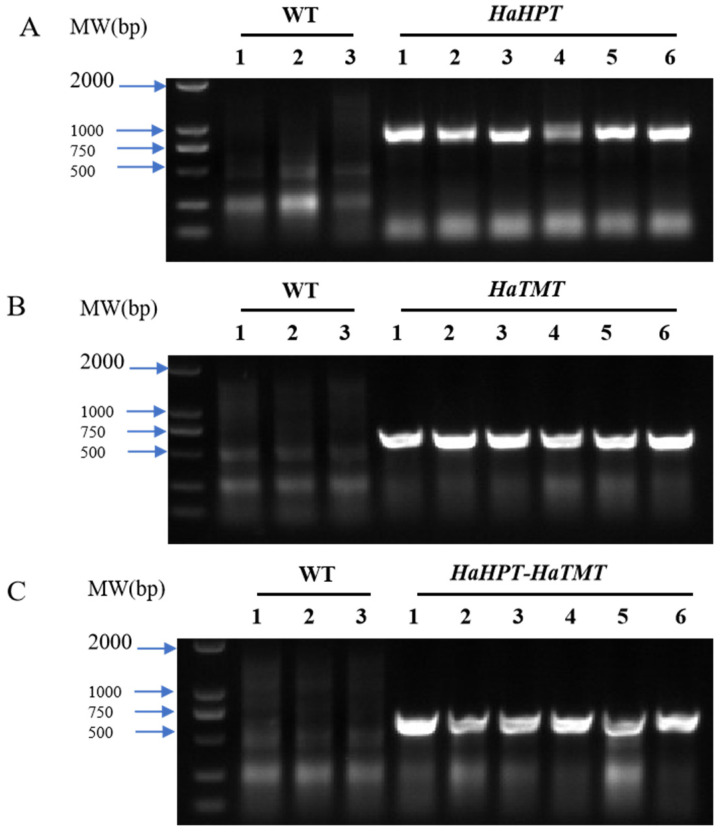
PCR analysis of transgenic plants. (**A**) PCR results of wild-type (WT) and *HaHPT* transgenic plants. (**B**) PCR results of WT and *HaTMT* transgenic plants. (**C**) PCR results of WT and *HaHPT*-*HaTMT* double-gene transformants. *HaHPT*-F/R primer was used to determine the introduction of *HaHPT* gene in (**A**). *HaTMT*-F/R primer was used to determine the introduction of *HaTMT* gene in (**B**,**C**). The different transgenic-positive plants (No. 1–3) from each construct in (**A**–**C**) were used in the follow-up phenotypic analysis. MW, molecular weight of markers (DL2000, Takara, Dalian, Liaoning, China).

In this study, *HaHPT* (GenBank No. XM_022148176.2) and *HaTMT* (EF495161.1), two sunflower genes involved in the synthesis of plant VEs, were transferred either alone or together into Ningjing 7 through genetic transformation (Figure 2). Forty transgenic rice plants of the T_0_ generation were obtained for each construct. T_1_ seeds were harvested from individual T_0_ plants. Primers designed to reside in the target genes and vector skeleton were designed for PCR analysis, and specific fragments could be amplified in all the transgenic rice tested (Figure 3). These specific fragments were further confirmed to come from sunflower *HaHPT* and *HaTMT* genes by Sanger sequencing. The results suggest that the foreign target genes have been integrated into the rice genome.

### 2.3. Relative Expression Analysis of Transgenic Plants 

**Figure 4:**
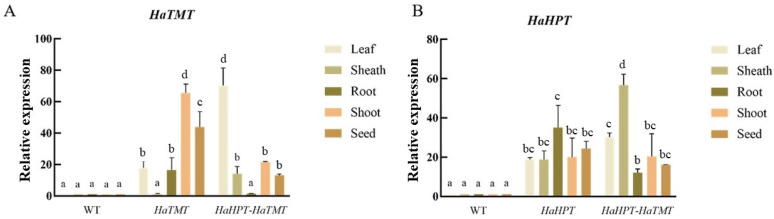
Relative expression of two genes in different tissues. (**A**) *HaTMT* gene expression difference between wild-type (WT) and transgenic plants. (**B**) *HaHPT* gene expression difference between wild-type (WT) and transgenic plants. For each transgenic construct, means ± SEMs from three different transgenic-positive lines are shown here. Statistical difference was determined by two-way (genotype and tissue) ANOVA with Tukey’s multiple comparison test. Means with no letter in common are significantly different at the 0.05 level.

Transgenic rice plants with positive PCR results were selected to extract total RNA from various vegetative tissues and mature seeds to detect the expression of introduced genes. The results (Figure 4) showed that while the expression of *HaHPT* or *HaTMT* is undetectable in the wild type, they are successfully expressed in the stems, leaves, and seeds of the transgenic plants.

### 2.4. Changes of Main Agronomic Traits of Transgenic Plants

**Figure 5:**
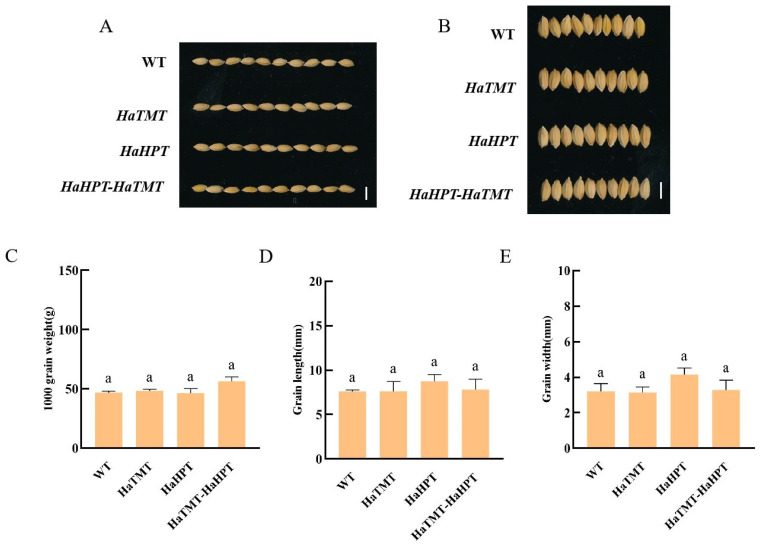
Comparison of phenotype between transgenic and wild-type (WT) seeds. (**A**,**B**) grain appearance quality, grain length (**A**) and grain width (**B**) of wild-type and *HaTMT*, *HaHPT*, *HaTMT-HPT* transgenic plants. Scale bar, 3 mm. (**C**–**E**) data of 1000-grain weight (**C**) grain length (**D**) and grain width (**E**) of wild-type and *HaTMT, HaHPT, HaTMT-HPT* transgenic plants. For each transgenic construct, means ± SEMs from three different transgenic-positive lines are shown here. Statistical difference was determined by one-way ANOVA with Dunnett’s multiple comparison test and WT was used as the control group. Means with the same lowercase letters indicate no significant difference from the control at the 0.05 level.

**Figure 6:**
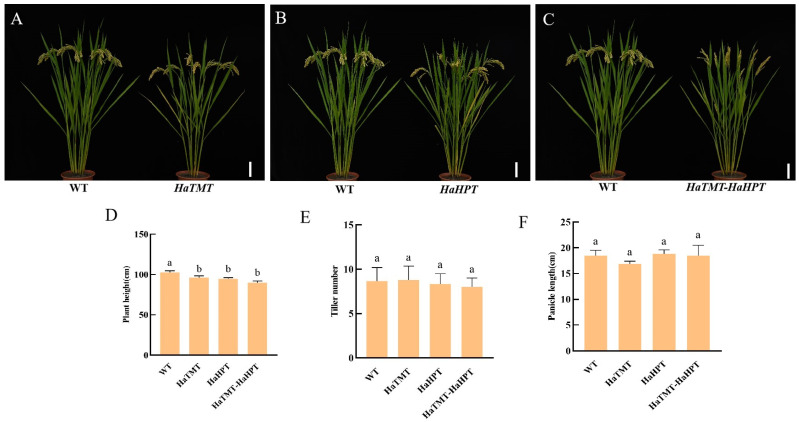
Comparison of plant traits between transgenic and wild-type (WT) plants. (**A**–**C**) plant morphology of wild-type and *HaTMT* (**A**), *HaHPT* (**B**), *HaTMT-HPT* (**C**) transgenic plants at the mature stage. Scale bar, 10 cm. (**D**–**F**) plant height (**D**), tiller number (**E**) and panicle length (**F**) for wild-type and *HaTMT*, *HaHPT*, *HaTMT-HPT* transgenic plants. For each transgenic construct, means ± SEMs from three different transgenic-positive lines are shown here. Statistical difference was determined by one-way ANOVA with Dunnett’s multiple comparison test and WT was used as the control group. Means with no letter in common are significantly different at the 0.05 level.

The agronomic traits of transgenic-positive plants were evaluated. At maturity, seeds were collected and tested for yield traits. It was found that there was no significant difference in grain length, grain width, 1000-grain weight (Figure 5), tiller number and panicle length between the transgenic plants and their wild type, but the plant heights of these transgenic plants were slightly reduced (Figure 6). Compared with the control, the plant heights of transgenic plants with *HaHPT*, *HaTMT*, and *HaHPT*-*HaTMT* constructs decreased by 7.79%, 6.17%, and 12.34%, respectively (Supplemental Table S2).

### 2.5. Vitamin E Content in Transgenic Plants

**Figure 7:**
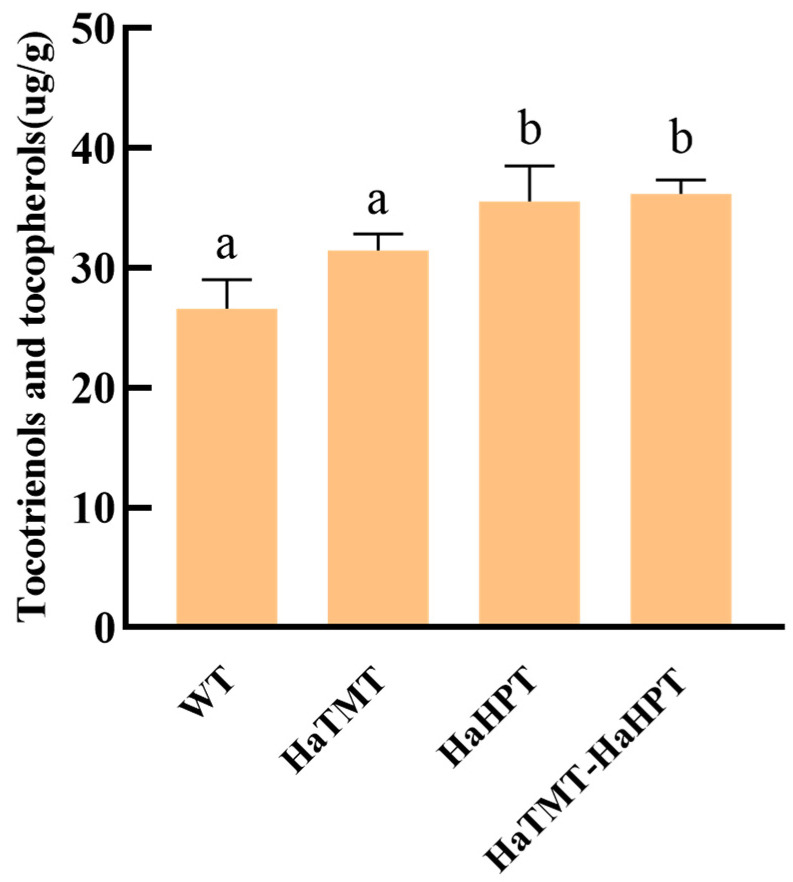
Contents of total vitamin E including tocopherol and tocotrienol in transgenic plant seeds (μg·g^−1^). For each transgenic construct, means ± SEMs from three different transgenic-positive lines are shown here. Statistical difference was determined by one-way ANOVA with Dunnett’s multiple comparison test and wild type (WT) was used as the control group. Means with no letter in common are significantly different at the 0.05 level.

The HPLC analysis of seed VE showed that the total VE contents of *HaHPT* alone and *HaHPT-HaTMT* double-gene transformants increased significantly, which were 1.34 times and 1.36 times higher than the wild type, respectively (Figure 7). However, the total VE content of *HaTMT* transgenic rice showed no significant difference compared with that of the wild type, which may be due to the fact that the *HPT* gene is located upstream of VE synthesis and functions as a key rate-limiting enzyme of the VE synthesis pathway. In addition, the total VE content of transgenic rice with the *HaHPT-HaTMT* double-gene transformant was significantly higher than that of any single-gene transformant.

**Figure 8:**
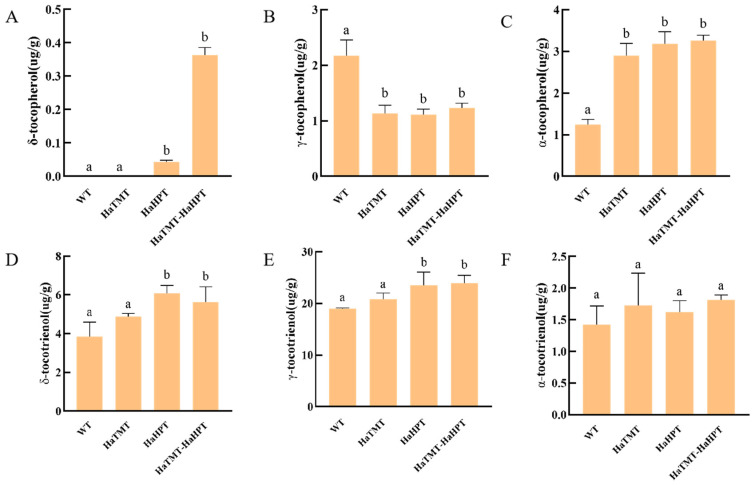
Different isomers of vitamin E (VE) in wild-type (WT) and transgenic seeds. All seeds were dehulled for the measurement of VE. (**A**–**C**) δ-T (**A**), γ-T (**B**), α-T (**C**) contents of wild-type and transgenic plants. (**D**–**F**) δ-3T (**D**), γ-3T (**E**), α-3T (**F**) contents of wild-type and transgenic plants. For each transgenic construct, means ± SEMs from three different transgenic-positive lines are shown here. Statistical difference was determined by one-way ANOVA with Dunnett’s multiple comparison test and WT was used as the control group. Means with no letter in common are significantly different at the 0.05 level.

The analysis of VE components in seeds showed that the α-tocopherol content of *HaTMT* transgenic plants was 2.33 times higher than that of wild-type plants. However, the γ-tocopherol content was significantly reduced to 50.24% of the wild type (Figure 8). The δ-tocopherol content of *HaHPT* transgenic plants showed a significant increase. The content of α-tocopherol increased 1.55 times. The content of γ-tocopherol was significantly decreased to 49.33% of the wild type. The content of δ-tocotrienol increased 1.6-fold. The content of γ-tocotrienol increased significantly and reached 1.25 times that of the wild type. The δ-tocopherol content of *HaHPT-HaTMT* transgenic plants also increased significantly. The α-tocopherol content increased 1.61 times, while the γ-tocopherol content decreased significantly to 54.66% of the wild type. The content of δ-tocotrienol increased 1.48 times, and the content of γ-tocotrienol also increased to 1.34 times that of the wild type.

### 2.6. Transcriptome and Expression Analysis

#### 2.6.1. Number of Differentially Express Genes (DEG) Statistics

**Figure 9:**
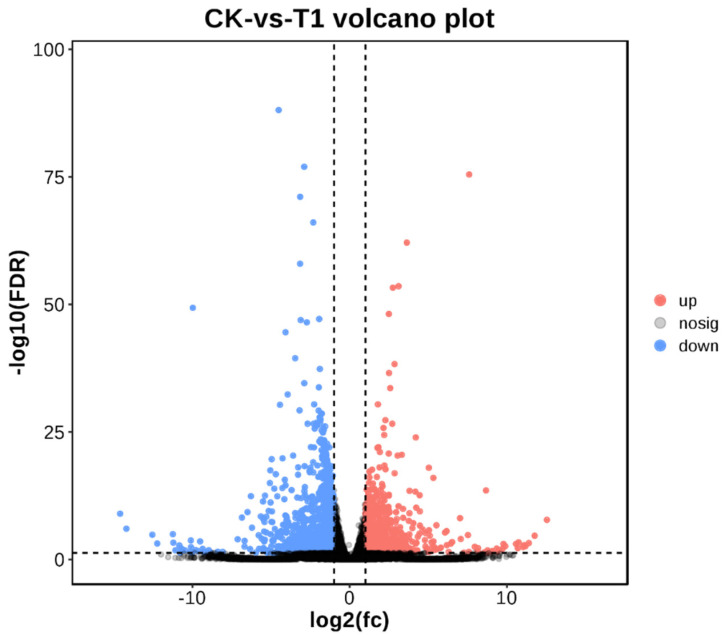
Volcano map of gene differential expression. CK indicates transgenic control, and T1 indicates representative *HaHPT-HaTMT* transformed plants for RNA-seq.

Compared with wild-type rice, 2345 differential genes were identified in *HaHPT-HaTMT* double-gene transformed plants (Figure 9). Among them, 832 genes were up-regulated, and 1513 genes were down-regulated. This suggests that the introduction of foreign genes leads to dramatic changes in gene expression.

#### 2.6.2. GO Enrichment Analysis of Differentially Expressed Genes

**Figure 10:**
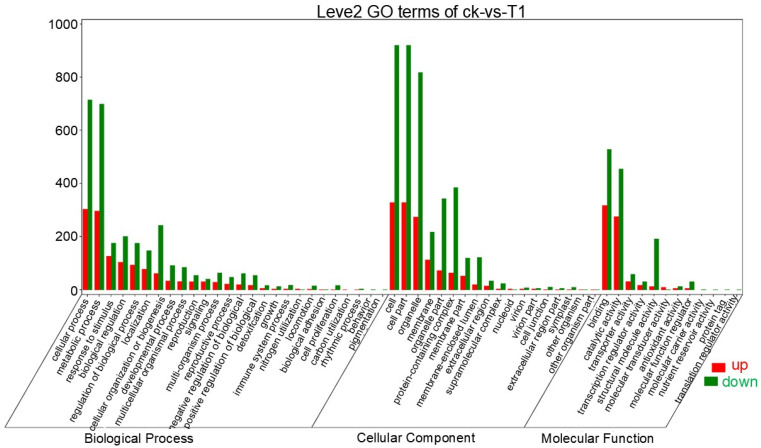
GO enrichment classification bar chart.

Gene Ontology (GO) comprehensively describes the properties of genes and gene products in organisms. According to the functional properties, the molecular function, cellular component, and biological process of genes were described. Compared with the wild-type plant, the expression of genes regulating the function of the three types of genes was mostly down-regulated in *HaHPT-HaTMT* double-gene transformed rice, resulting in an increase in the number of differential genes (Figure 10).

**Figure 11:**
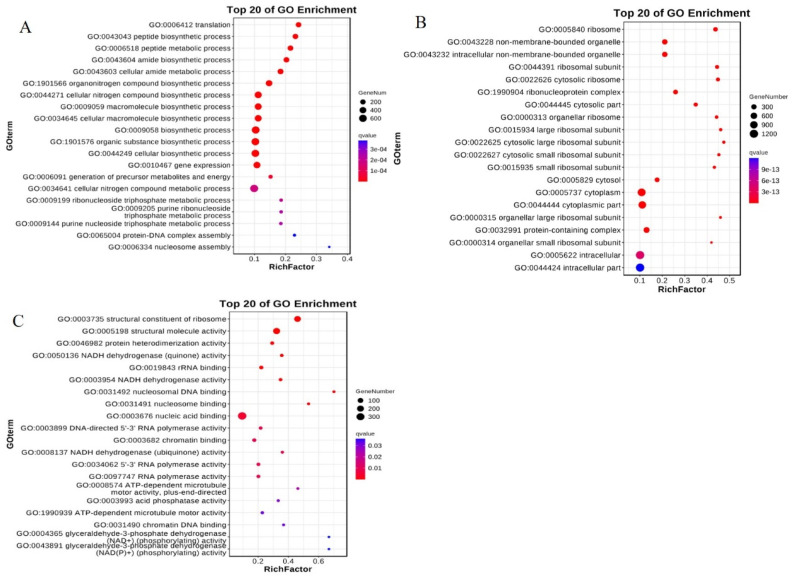
Gene Ontology (GO) enrichment bubble diagram. (**A**) Bioprocess GO enrichment bubble map. (**B**) GO enrichment bubble map of cell components. (**C**) GO enrichment bubble map of gene molecular function. The top 20 GO terms with the smallest Q value are used as GO enrichment bubbles. The ordinate represents the GO term, and the abscissa represents the enrichment factor (the number of differentially expressed genes in the GO term is divided by all the numbers in the GO term), and the size represents the number of genes. The redder the color, the smaller the Q value.

In terms of the biological processes involved, the top annotated processes were translation, peptide biosynthesis, peptide metabolism, and amide biosynthesis, cellular amide metabolism, organonitrogen compound biosynthesis, cellular nitrogen compound biosynthesis, macromolecule biosynthesis, cellular macromolecule biosynthesis, and the biosynthetic process (Figure 11A); thus, they were mostly enriched in molecular synthesis processes. In terms of cellular components, the most-enriched were ribosomes, non-membrane-bound organelles and intracellular non-membrane-bound organelles, ribosomal subunits, cytosolic ribosomes, etc. (Figure 11B), and most of the enrichment component was in the process of ribosome synthesis. In terms of the molecular function of the differential genes, the most-enriched genes included the structural component of ribosome and NADH dehydrogenase (quinone) activity, glyceraldehyde-3-phosphate dehydrogenase, (NAD+) (phosphorylating) activity, (NAD(P)+) (phosphorylating) activity, etc. (Figure 11C). These results suggest that exogenous *HaHPT-HaTMT* transforming in rice mainly regulates the metabolic process and affects the synthesis of VE in grains.

#### 2.6.3. KEGG Enrichment Analysis of Differentially Expressed Genes

**Figure 12:**
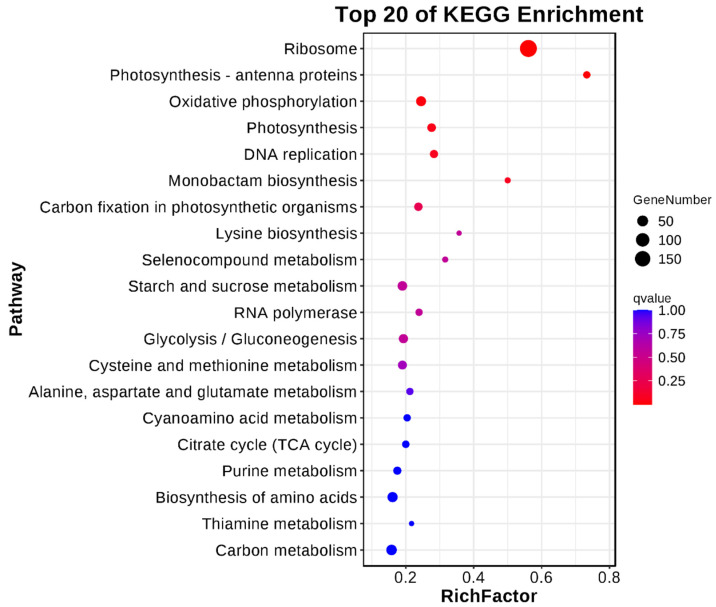
KEGG (Kyoto Encyclopedia of Genes and Genomes) enrichment bubble diagram. The top 20 pathways with the smallest Q value are shown here. The ordinate is the pathway, and the abscissa is the enrichment factor (the number of differential genes in the pathway is divided by the number of all genes in the pathway), and the size indicated the number. The redder the color, the smaller the Q value.

KEGG (Kyoto Encyclopedia of Genes and Genomes) analysis can identify the major biochemical and signaling pathways involved in the differential genes and enable us to further understand their biological functions. It was found that the ribosome pathway had the lowest Q value and the largest number of enriched differential genes. In addition, there were different expressions of differential genes in the photosynthesis–antenna protein pathway, oxidative phosphorylation pathway, photosynthesis pathway, and DNA replication pathway (Figure 12).

**Figure 13:**
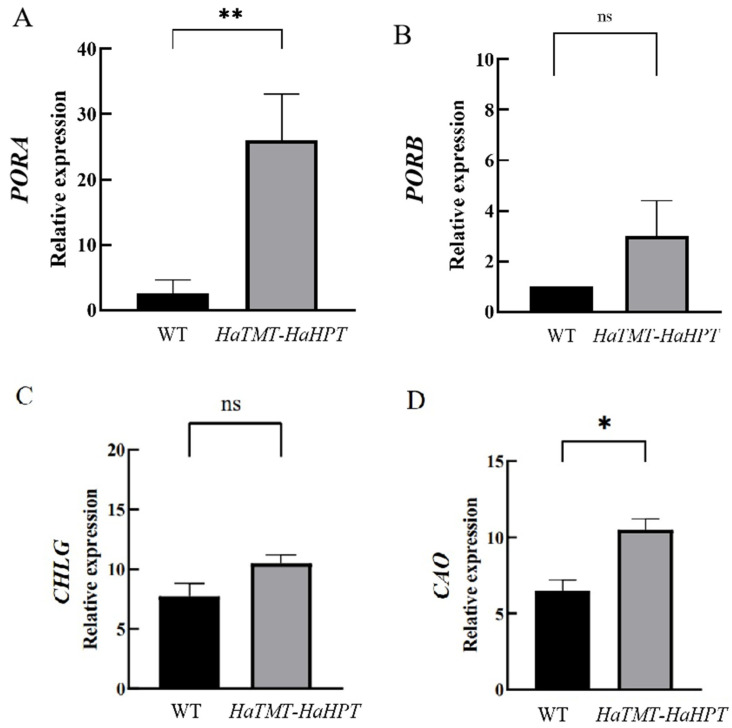
Relative expression of genes related to chlorophyll synthesis pathway. (**A**) *protochlorophyllide reductase A* (*PORA*) gene expression difference between wild-type and transgenic rice. (**B**) *protochlorophyllide reductase B* (*PORB*) gene expression difference between wild-type and transgenic rice. (**C**) *chlorophyll synthase gene* (*CHLG*) gene expression difference between wild-type and transgenic rice. (**D**) *chlorophyllide a oxidase* (*CAO*) gene expression difference between wild-type and transgenic rice. Analysis of difference significance is based on student’s *t*-test, * *p* < 0.05, ** *p* < 0.01, ns: No significance.

Studies have shown that phytol derived from chlorophyll degradation in *Arabidopsis* is an important precursor for tocopherol synthesis [20]. Some experiments have also shown that several genes involved in the chlorophyll pathway affect tocopherol levels. For example, the chlorophyll synthase gene *CHLG* in *Arabidopsis* was shown to regulate tocopherol synthesis [21]. In addition, two POR genes encoding prochlorophyll ester reductase were reported to involve the chlorophyll pathway and affect tocopherol content in maize (*Zea mays*) seeds [22]. In this study, genes related to the chlorophyll synthesis pathway were further detected by reverse transcription-polymerase chain reaction (RT-PCR) analysis. They included *protochlorophyllide reductase A* (*PORA*, LOC_Os04g58200), *protochlorophyllide reductase B* (*PORB*, LOC_Os10g35370), *CHLG* (LOC_Os05g28200), and *chlorophyllide a oxidase* (*CAO*, LOC_Os10g41760). The results showed that the expression of four genes in the seeds of transgenic plants was moderately or significantly higher than the wild type (Figure 13), consistent with the RNA-seq analysis.

## 3. Discussion

In the synthesis of tocopherol, HGA and PPP are catalyzed by HPT to form MPBQ. Then, under the action of 2-methyl-6-phytylbenzoquinol methyltransferase (MPBQ-MT), MPBQ produces 2,3-dimethyl-5-phytylbenzoquinol (DMPBQ). DMPBQ can then generate δ-tocopherol and γ-tocopherol under the catalyzation of tocopherol cyclase (TC). Finally, under the action of γ-TMT, the above two substrates are converted into β-tocopherol and α-tocopherol, respectively [23,24,25]. In general, the key genes in the VE synthesis pathway include *HPT*, *TC*, *MPBQ-MT* and *γ-TMT*. Most of these genes have been cloned, and their roles in the synthetic pathway of VE have been identified [26,27,28]. 

Previous studies have shown that the use of synthetic biology and transgenic technology can enhance nutrient metabolism. For example, Zhu et al. [7] created a novel “ame-thyst rice” germplasm for astaxanthin synthesis in rice endosperm by using a multi-gene vector system. In addition, increased α-tocopherol levels were shown in the stable transformants of tobacco species including *Nicotiana tabacum* [29,30] and *Nicotiana benthamiana* by transiently expressing *Arabidopsis* TC and HPT [31]. Similarly, the *Agrobacterium*-mediated transformation of *indica* rice ASD16 with *TC* and *HPT* showed increased VE (mainly α-tocopherol) content [32]. In general, increasing the content of metabolites requires the regulation of the entire pathway, and rate-limiting enzymes involved in the synthetic process usually play an essential role in the pathway. In the biosynthesis of VE, our study showed that the *HPT* gene acted as a rate-limiting enzyme to control the synthesis of VE, while *γ-TMT* was a key gene that converted γ-tocopherol to α-tocopherol. Studies have shown that the overexpression of the *HPT* gene can significantly increase tocopherol levels in *Arabidopsis* [18], and the overexpression of the *ZmTMT* gene in maize has markedly increased α-tocopherol levels [33]. Recently, another study cloned a gene (*ZmPORB1*) that positively regulated tocopherol content in maize grains. The overexpression of *ZmPORB1* significantly increased tocopherol levels, while the knockout of the gene showed the opposite effect [34]. Thus, *PORB1*, in combination with the genes used in this study, can provide more target genes for bioengineering to improve the VE content of crop grains in the future.

In this study, two key genes, *HaHPT* and *HaTMT*, which affect the synthesis of plant VE, were transferred into Ningjing 7. After the seeds matured, the representative transgenic plants were selected, and the seeds per plant were collected. Compared with the wild type, the content of VE in the seeds of transgenic plants obtained under different transgenic combinations increased significantly. The total content of VE in transgenic *HaTMT* plants increased by 18.23%, while VE in transgenic *HaHPT* plants increased by 33.26% compared to the wild type. At the same time, the content of VE in transgenic *HaTMT*-*HaHPT* plants increased by 35.98%, which was the most obvious in all transgenic events. The VE isomers of these transgenic combinations were different except for α-tocotrienol. The results showed that the double-gene transformants had remarkable effects in both increasing the total VE content and changing the VE composition. We found the total VE content in either *HaHPT* transgenic plants or *HaTMT*-*HaHPT* transgenic plants increased greatly, which might be due to the use of the constitutive strong promoter *ZmUbi* to drive the *HaHPT* gene, resulting in the accumulation of VE in grains. In addition, these transgenic combinations did not affect agronomic traits such as the 1000-grain weight, tiller number and panicle length, consistent with the previous study [32]. However, compared with the wild type, the flowering time of the *HaHPT-HaTMT* transgenic rice plants was 2–3 days later, so there was little difference in the panicle architecture between the wild type and transgenic plants (Figure 6). Transcriptome analysis showed that the genes involved in the ribosome pathway and photosynthesis pathway (including *PORA*, *PORB*, *CHLG* and *CAO*) were up-regulated. The former pathway might provide energy, while the latter might promote precursor synthesis for VE synthesis, as described previously [34].

In conclusion, our study demonstrates that the heterologous expression of *HaTMT* from sunflower in rice promotes the accumulation of α-tocopherol in rice grains, while the heterologous expression of *HaHPT* results in an increase in the total VE content in rice grains. Moreover, the transgenic background of Ningjing 7 is noteworthy—it is a high-yield *japonica* variety widely cultivated in mid–lower Yangtze River regions. This study demonstrates the feasibility of breeding rice cultivars with high VE content through metabolic engineering and provides a valuable germplasm for this purpose.

## 4. Materials and Methods

### 4.1. Plant Materials

*Japonica* rice Ningjing 7 was used as the genetic background for the rice transformation experiments.

### 4.2. Strains, Carriers and Main Reagents

*Agrobacterium* strain EHA105 was purchased from Nanjing Qingke Biotechnology Co., Ltd. (Nanjing, Jiangsu, China). The vector backbone was preserved and provided by our laboratory. Restriction endonuclease and T_4_ DNA ligase were purchased from New England Biolabs (NEB) Co., Ltd. (Beijing, China). PCR primers and quantitative primers were synthesized by Nanjing GenScript Co., Ltd. (Nanjing, Jiangsu, China). Quantitative RT-PCR analysis was performed using the TB Green^®^ Premix Ex Taq™ kit from TaKaRa Biological Company (Cat # RR420A, Dalian, China) and the ABI prism 7900 quantitative RT-PCR system. The standard samples of tocopherol and tocotrienol isomers were purchased from Shanghai Huicheng Biological Company (Shanghai, China). The common chemical reagents were purchased from Sinopharm Chemical Reagent Co., Ltd. (Beijing, China).

### 4.3. Primer Design

The specific primers were designed using the primer design website (https://www.ncbi.nlm.nih.gov/tools/primer-blast/index.cgi) (accessed on 10 October 2021), and Gramene (http://www.gramene.org/, accessed on 10 October 2021) was used to detect the primer specificity. The primers were synthesized by Nanjing GenScript Co., Ltd. and used for the subsequent amplification of the target gene fragment and sequencing.

### 4.4. Vector Construction

Using Goldengate technology [35], the three standard elements (promoter, CDS and terminator) of the two target genes *HaHPT* and *HaTMT* were loaded into the zero-level vector, respectively. Each standard element contained B*sa*I and B*bs*I restriction sites at both ends, and the target gene did not contain these two restriction sites. The three standard elements of each target gene were recombined into the first-level vectors, and then the two first-level vectors were recombined into the second-level vector. Finally, the final secondary vectors were introduced into *calli* induced from Ningjing 7 mature seeds by *Agrobacterium*-mediated transformation [36].

### 4.5. Identification of Transgenic Rice

Leaf DNA was extracted by the CTAB method [37]. The primers for PCR analysis were *HaHPT*-F/R and *HaTMT*-F/R, which were used to detect *HaTMT*, *HaHPT*, and *HaTMT-HaHPT* transgenic plants, respectively (Supplemental Table S1). 

### 4.6. qRT-PCR Analysis

Quantitative PCR primers were designed using the GenScript’s website (https://www.genscript.com/tools/real-time-pcr-taqman-primer-design-tool) (accessed on 11 October 2022) (Supplemental Table S1). The *UBQ* gene (*Ubiquitin*, LOC_Os01g22490) was used as an internal control, and three replicates were set for each sample to detect the expression of the target genes. Total RNA was extracted from wild-type and transgenic plants in different tissues. RNA was extracted according to the protocol of the RNA Prep Pure Plant kit (TIANGEN Biotechnology). Total RNA (2 μg) was synthesized with SuperiorScript Reverse Transcriptase (Enzynomics) and oligo(dT) primers for single-stranded cDNA. A PikoReal real-time PCR system (ThermoFisher Scientific, Shanghai, China) with TB Green^®^ Premix Ex Taq™ (Tli RNaseH Plus) (Cat # RR420A) was used for qRT-PCR. The 2^−ΔΔCT^ method was used to analyze and process qRT-PCR data [38,39].

### 4.7. Extraction of Vitamin E

Homozygous transgenic-positive rice seeds were identified by consistent Hygromycin B resistance. The husk of the seeds was removed by a dehusker (Taizhou Grain Instrument Factory, JLGJ4.5 model, Taizhou, China). The vibratory ball mill (POWTEQ Company, GT300 model, Beijing, China) was used to grind the brown rice for the determination of VE content. About 2–3 g brown rice flour was weighed and extracted by the Soxhlet extraction method. The fat was extracted by referring to Chinese National Standard GB 5009.82-2016 [40]. After the crude fat was extracted and dried, the oil–aluminum cup was washed with 2 mL n-hexane. The solution was transferred to a 10 mL plastic calibration tube with a pipette gun, and the step was repeated again. The oil cup was washed with 2 mL n-hexane, and then n-hexane was added to a final volume to 5 mL. After mixing evenly, the solution was filtered with a 0.22 μm organic filter membrane, and 1 mL of filtrate was transferred into a brown sample bottle with a capacity of 1.5 mL. The samples were sealed with a Teflon cover and stored at −20 °C before loading. 

### 4.8. HPLC Analysis

Ultra-high-performance liquid chromatography (Thermo, UltiMate 3000, Shanghai, China) was performed on the public technology platform of the State Key Laboratory of Crop Genetics and Germplasm Enhancement and Utilization. The column was an HSS T3 of 1.8 μm, 2.1 × 50 mm; the mobile phase was methanol/water 95:5 (*v*/*v*); the flow rate was 0.2 mL·min^−1^; and the injection amount was 2 μL.

### 4.9. Transcriptome Analysis

The immature seeds from Ningjing 7 on the ninth day after flowering and its HaHPT-HaTMT transgenic plants were rapidly placed in liquid nitrogen and then transferred to a −80 °C refrigerator for storage. The glume-removed seeds were ground during the filling stage and sent to Kidio Biotechnology Co., Ltd. (Wuhan, Hubei, China). for transcriptome analysis. The criteria for screening DEGs were as follows: |log2Ratio| ≥ 1 and *q*-value ≤ 0.05. GO functional analysis was performed using Blast2go version 5 [41]. The KEGG database [42] was used for pathway enrichment analysis. For each sample, two replications were performed. At least 5G clean reads were obtained for each sample. All requests for RNA sequencing reads will be provided in a timely manner.

### 4.10. Statistical Analysis

GraphPad Prism 8.0.2 (GraphPad Software, Boston, MA, USA) was used for the analysis of variance (ANOVA) with a significance level of 0.05. Microsoft Excel 2019 for Windows was used for the Student’s *t*-test.

## Data Availability

Data are contained within the article and Supplementary Materials.

## Supplementary Materials

The following supporting information can be downloaded at: https://www.mdpi.com/article/10.3390/plants13172392/s1, Table S1: PCR primers used for the identification of exogenous gene insertion and expression; Table S2: Agronomic trait comparison of wild-type (WT) and transgenic plants.
