# Supplementary material for: Heterologous Expression of Sunflower HaHPT and HaTMT Genes Enhances Rice-Grain Vitamin E Content
Source: Plants (Basel). 2024 Aug 27;13(17):2392. doi: 10.3390/plants13172392 (PMC11397534; doi:10.3390/plants13172392)
Supplement: Supplementary file 1 [file plants-13-02392-s001.zip › plants-3086685-supplementary.pdf]

Supplemental Table S1. PCR primers used for the identification of exogenous gene insertion and expression.

Gene	Forward sequence (F)(5′-3′)	Reverse sequence (R)(5′-3′)	Purpose	GenBank No.
UBQ	AACCAGCTGAGGCCCAAGA	ACGATTGATTTAACCAGTCC	Control of expression	AK061988
HaHPT	TCGGTCTCAAATGAGTCATATAACA	GTGGTCTCAAAGCTTAGAATCTTAAT	Transgenic detection and sequencing	XM_022148176.2
HaTMT	TTGGTCTCAAATGGCTACGACG	AAGGTCTCAAAGCTTAGCCCTTATCA	Transgenic detection and sequencing	EF495161.1
qHaHPT	TTTCTTTGCTAGCCGTGCAG	AACTGAAAGTTCGCCGGATG	Expression analysis	
qHaTMT	AACGTTCGCCTCTGTTTCAG	GAAACCTTATCGGCCAACCC	Expression analysis	

Supplemental Table S2. Agronomic trait comparison of wild type (WT) and transgenic plants.

Agronomic traits	WT	*HaTMT*	*HaHPT*	*HaTMT-HaHPT*
Plant height/cm	102.67±1.70	96.33±1.70*	94.67±1.25**	90.00±1.63**
Number of tillers	9.33±1.05	6.33±0.47	8.33±0.94	7.00±1.63
Grain length/mm	7.61±0.13	8.63±1.38	8.75±0.62	6.81±1.06
Grain width/mm	3.20±0.37	3.47±0.69	4.15±0.30	2.95±0.79
Panicle length/cm	18.50±0.82	16.83±0.47	18.83±0.62	18.50±1.63
Seed-setting rate	88.47%±3.84	89.12%±3.61	93.01%±2.72	88.28%±3.03
1 000-grain weight/g	53.43±0.49	48.50±1.03	48.17±0.95	59.84±1.50

Data are mean ± standard deviation. Analysis of difference significance is based on Student's *t*-test. *P <0.05, **P <0.01.
